# The added value of frequent physical activity group sessions in a combined lifestyle intervention: A cluster randomised trial in primary care

**DOI:** 10.1016/j.pmedr.2020.101204

**Published:** 2020-09-11

**Authors:** Brenda A.J. Berendsen, Marike R.C. Hendriks, Geert M. Rutten, Stef P.J. Kremers, Hans H.C.M. Savelberg, Nicolaas C. Schaper

**Affiliations:** a Nutrition and Movement Sciences, Maastricht University Medical Centre, PO Box 616, 6200 MD Maastricht, the Netherlands; b Maastricht University Campus Venlo, Venlo, the Netherlands; c Health Promotion, Maastricht University Medical Centre, Maastricht, the Netherlands; d Internal Medicine, Maastricht University Medical Centre, Maastricht, the Netherlands

**Keywords:** CLI, Combined lifestyle intervention, GP, General practitioner, HCC, Health care cluster, HCP, Health care professional, LSC, Lifestyle coach, MVPA, Moderate to vigorous physical activity, PA, Physical activity, Combined lifestyle intervention, Primary care, Health promotion, Dose response, Physical activity, Overweight

## Abstract

Combined lifestyle interventions (CLIs) that target both physical activity (PA) and diet have been shown to improve PA and health of adults who are overweight; however, optimal amount of guidance remains unclear. This study evaluated the effects of adding PA group sessions to a standard CLI in primary care in the Netherlands. 411 participants (BMI 34.5 ± 4.4 kg/m^2^) in thirty locations were randomised into a one year CLI or CLI+. CLI comprised individual meetings with lifestyle coach (LSC) and physiotherapist, and group meetings with dietician. PA group sessions were added in CLI+. Primary outcome was minutes of moderate to vigorous PA per week. Secondary outcomes of PA, diet and cardiovascular risk factors (i.e. waist circumference, HbA1c and blood pressure) were evaluated after 12 and 24 months with multilevel analyses. Data were collected from 2010 to 2015. Significant between-group effects were only found for waist circumference, which was significantly lower at twelve months in the CLI+ group (p = 0.011), no other between-group differences were found. Several within-group changes were significant. After participating in the intervention, walking time increased with 83 ± 35 min/week and 100 ± 38 min/week, and BMI decreased with 0.7 ± 0.3 kg/m^2^ and 1.5 ± 0.3 kg/m^2^ in CLI and CLI+ respectively (p < 0.001). Diet, HbA1c and systolic blood pressure changed favourably in both groups. Adding PA group sessions did not elicit added, sustained benefits. A programme with individual meetings with LSC and physiotherapist, and group meetings with dietician can be sufficient to facilitate a healthy lifestyle and reduce cardiovascular risk factors in a population with overweight.

## Highlights

• A combined lifestyle intervention improves lifestyle in overweight and obese subjects.
• Real-world setting study shows that changed lifestyle after a combined lifestyle intervention can be sustained.
• Adding group sessions with a physiotherapist had no added value.

## 1 Background

A lifestyle consisting of moderate physical activity and dietary discretion is a clear opportunity to decrease health risks associated with overweight; however, the adoption of health enhancing behaviours remains challenging. In 2018 in the Netherlands, 35% of adults were classified as overweight (BMI 25–30 kg/m^2^), and 15% as obese (BMI > 30 kg/m^2^) (CBS, 2018). In addition, it has been estimated that 56% of the Dutch population does not meet the daily activity guidelines (Gezondheidsraad. Beweegrichtlijnen, 2017). By targeting physical activity (PA), the negative consequences of overweight can be prevented directly and indirectly, as evidence shows that an increase of PA can result in health benefits even in absence of weight loss (Ekelund et al., 2007).

Many efficacy trials of combined lifestyle interventions, targeting both PA and diet, have been performed in a controlled research setting (Knowler et al., 2002, Knowler et al., 2009, Look Ahead Research Group, 2010), limiting practical generalisability. In primary care settings, practice nurses have been suggested to function as a lifestyle coach (LSC) due to their expertise, contact with general practitioner (GP) and potential reach (Goodpaster et al., 2010, Hardcastle et al., 2008, Vermunt et al., 2012, Driehuis et al., 2012). Recently, in the Netherlands a programme delivered by LSCs showed favourable changes in psychosocial determinants, behaviour and weight (van Rinsum et al., 2018). Yet, because many intervention trials were performed in researcher controlled settings and so few intervention trials have been executed in primary care settings (Knowler et al., 2002), the question remains how much guidance is required and feasible in daily practice. High dose guidance programmes (e.g. the four-year Look AHEAD intervention with high dose guidance in the first year (Wadden et al., 2011) show beneficial results in improving lifestyle behaviours, reducing overweight and thereby potentially improving health, compared to a group receiving no or very little intervention (e.g. one consultation or information only) (Goodpaster et al., 2010, Look Ahead Research Group, 2014). However, feasibility of such high dose programmes in actual practice is questionable, due to the required time investments by health care professionals and participants, as well as financial issues (Berendsen et al., 2015). Moreover, studies have also identified specific challenges of a multicomponent intervention, and a ceiling effect might occur (Hardcastle et al., 2008, Driehuis et al., 2012). Therefore, implementation of lower dose lifestyle interventions in practice might be preferable. Additionally, research by Fraser and colleagues (Fraser and Spink, 2002) and Kwak and colleagues (Kwak et al., 2006) suggested that adding group sessions rather than individual meetings would improve adherence and effectiveness of the intervention via group cohesion; and it might influence two basic needs for autonomy and long term effects: relatedness and competence (Deci and Ryan, 2000, Rutten et al., 2014). Unfortunately, the optimal dose and characteristics of PA counselling are still unclear. Interventions of shorter duration and/or less guidance than e.g. the Look AHEAD trial yielded relevant effects on lifestyle and weight after six to twelve months of guidance (Goodpaster et al., 2010, Hardcastle et al., 2008, van Rinsum et al., 2018), while others showed small or even no effects after 2.5 to three years of guidance (Vermunt et al., 2012, Driehuis et al., 2012).

In summary, less guidance might be more feasible in real life settings, but may be less effective in improving lifestyle (Wadden et al., 2011). Therefore, we performed a cluster randomised controlled trial, integrated in daily practice, in subjects who were overweight or obese. We compared the effects on lifestyle behaviour and cardiovascular risk factors of a standard combined lifestyle intervention (CLI) with a combined lifestyle intervention with additional group sessions led by a physiotherapist (CLI+). CLI and CLI+ had equal guidance by a LSC and dietician, but differed in terms of number of group sessions under guidance of a physiotherapist (Berendsen et al., 2011). Guidance of both CLI and CLI+ were based on Self-Determination Theory and utilised Motivational Interviewing (MI) (Deci and Ryan, 2000).

We hypothesised that participants of both the CLI and CLI+ would show beneficial changes in lifestyle and cardiovascular risk factors, and that the additional group sessions with the physiotherapist of CLI+ would lead to improved PA behaviour compared to CLI. Furthermore, we expected additional beneficial effects on diet and cardiovascular risk factors in CLI+ compared to CLI.

## 2 Methods

### 2.1 Design

The design of the study has been described in detail elsewhere (Berendsen et al., 2011). CLI and CLI+ were offered by cooperations of GPs, LSCs, physiotherapists and dieticians, collectively called Health Care Clusters (HCCs). Thirty HCCs with experience in combined lifestyle interventions were cluster randomised into CLI or the CLI+ with a computerised random number generator after being matched pair wise based on HCC size and urban/rural area by one of the principal investigators. Cluster randomisation reduced the risk of contamination between participants and the risk of bias at the level of the professionals. As these professionals knew about the differences between CLI and CLI+ before the trial, blinding was not possible. Participants were not aware of the allocation of their HCC to CLI or CLI+. The interventions lasted one year with an additional year of follow-up in order to determine their sustainability.

The study was approved by the Medical Ethics Committee of the Maastricht University Medical Centre. All participants gave informed consent.

### 2.2 Participants

Inclusion criteria for both CLI and CLI+ were (1) a BMI between 25–35 kg/m^2^, combined with at least one of the following serious related comorbidities: sleep apnoea, arthritis, cardiovascular disease and/or type 2 diabetes; or (2) a BMI between 35–40 kg/m^2^, but without these related serious comorbidities. In addition, participants should fail to meet the Dutch norm for healthy PA (30 min of moderate to vigorous PA (MVPA) on at least five days a week), and had to be sufficiently motivated to change their PA level and dietary behaviour. To assess motivation, the LSA had a first appraisal of participants’ PA pattern and motivation by showing propositions to the participant and asking which most applied to their situation (e.g. ‘I am currently not physically active and I do not intend to become physically active’ or ‘I am currently not physically active, but I am considering to change this’). A detailed sample size calculation has been described earlier, resulting in a projected sample of 600 participants, with a power of 80% and significance level of 5%, accounting for 5% intra-cluster correlation, 30% drop-out of participants and entire HCCs (Berendsen et al., 2011).

### 2.3 Interventions

Both programmes comprised guidance of one year; an elaborate description has been published earlier (Berendsen et al., 2011) and observed dose has been described in an extensive process evaluation (Berendsen et al., 2015). The amount and type of guidance by the physiotherapist differed between the programmes. CLI and CLI+ included six individual meetings of 30 min with the physiotherapist, whereas guidance by the physiotherapist in CLI+ included an additional 26–34 group sessions of an hour. The group sessions of CLI+ took place in the first four months and consisted of physical exercise to overcome barriers and increase physical capacity. The individual consultations in CLI and CLI+ with the physiotherapist in both programmes were aimed at identifying barriers to PA and drawing up a plan to remain physically active without the supervision by health care professionals (HCPs).

The amount of guidance by the LSC and dietician was similar in the two programmes; six individual meetings with the LSC, and three individual meetings and seven group meetings with the dietician (all 25 min each). All HCPs in the team were trained to use MI and goal setting to facilitate behaviour change and maintenance (Miller and Rollnick, 1991, Helmink et al., 2010). In both programmes, the LSC had a key role in supporting the participants and discussed progress and barriers of behavioural change. Meetings with the dietician consisted of nutritional recommendations, education, coping with high-risk situations, checking dietary behaviour and interaction between participants, based on guidelines for diabetes and overweight (Kwaliteitsinstituut voor de Gezondheidszorg, 2008, Federatie and Voedingsrichtlijn, 2006).

### 2.4 Outcomes

The primary outcome was self-reported MVPA in minutes per week. In addition, total metabolic equivalent (MET) minutes, walking and sitting time were included because a behavioral, compensatory effect might occur when MVPA increases (Driehuis et al., 2012, Helmink et al., 2013). The PA outcomes were measured with the short version of the International Physical Activity Questionnaires (IPAQ); reliability tests showed a correlation coefficient of 0.75 (Craig et al., 2003), reinforced by other reliability studies (van Poppel et al., 2010). The IPAQ was self-administered every six months (at baseline, six months, twelve months, 18 months and 24 months). Outcomes were calculated according to the IPAQ protocol (IPAQ Group, 2005). An additional self-administered question assessed whether participants adopted an independent physical exercise activity (i.e. exercise besides the guidance by the physiotherapist) after one and two years. In contrast to the study design (Berendsen et al., 2011), questionable user friendliness (Berendsen et al., 2014) and low number of usable measurements prevented evaluation of PA using activity monitors.

Diet was operationalized as weekly consumption of fruit, vegetables, snacks and candy, based on the dietary guidelines that were used in the interventions (Kwaliteitsinstituut voor de Gezondheidszorg, 2008, Federatie and Voedingsrichtlijn, 2006), and assessed using the self-administered ENVET. The ENVET consisted of questions regarding fruit and vegetable consumption, with agreement of 0.35 for vegetable consumption and 0.51 for fruit consumption validated with diary records (van Assema et al., 2002). Participants indicated the number of days per week they consumed fruit, and how many they would typically have on a day; those two values were multiplied to calculate weekly fruit consumption. Vegetable consumption was measured and calculated with the same procedure, but with number of servings. The other items were based on the Fat-list, which is a valid method to classify subjects (van Assema et al., 2001). Participants indicated number of occasions of snack and candy consumption per week. Reliability and validity was not assessed for snack or candy consumption independently.

Cardiovascular risk was operationalized as body composition, blood pressure and blood lipids, and assessed by the local HCP at baseline, after one and two years. Body composition outcomes were height, weight, waist circumference and fat percentage. Fat percentage was assessed with a tetrapolar bioelectrical impedance device (OMRON BF511). Further, blood pressure was measured and blood samples were taken to assess HbA1c (mmol/mol), total cholesterol (mmol/L), HDL (mmol/L) and creatinin (µmol/L).

### 2.5 Analyses

Differences in baseline characteristics, rate of drop-out and adverse events between the two programmes were assessed with t-tests or Chi-square tests. Descriptive statistics and Chi-square tests evaluated whether participants adopted an independent activity and whether participants complied with the Dutch PA norm.

Linear mixed model analysis techniques were applied to the longitudinally measured primary and secondary outcomes. The analyses involved a three level design with repeated measures as the first level (AR1 covariance structure for serial correlation), participant as second level (unstructured covariance) and HCC as third level (unstructured covariance). Changes compared to baseline were assessed with pairwise comparisons, applying the Bonferroni correction. Primary analyses were performed with MVPA, walking and sitting time and MET-minutes measured by IPAQ as dependent variables. Independent variables were moment of measurement and programme (CLI and CLI+) and moment of measurement*programme; covariates were age, gender, BMI at baseline and season. Secondary analyses were performed with the other PA outcomes, dietary behaviour, BMI, weight, waist circumference, fat percentage, HbA1c, cholesterol, HDL and blood pressure as dependent variables. Linear mixed model analyses account for data missing at random, without imputation of missing data (Twisk and de Vente, 2002). Exploratory analyses were done using the per protocol principle in which participants were excluded who were registered as dropout via HCP or via communication with researchers. Analyses were done in SPSS 21.0 with a significance level of 0.05, unless mentioned differently.

## 3 Results

### 3.1 Baseline characteristics

411 participants were included, of which 164 in CLI (from 14 HCCs) and 247 in CLI+ (from 15 HCCs). Mean age was 55.1 ± 12.4 years, 35.3% was male and mean BMI was 34.5 ± 4.4 kg/m^2^. At baseline, demographics did not differ between the two study groups (Table 1) (Berendsen et al., 2015). On average, participants in CLI+ had higher values of MVPA time (p = 0.032) and total PA (p = 0.030). Main analyses were corrected for these baseline differences; change from baseline was used as outcome for the multilevel analyses of MVPA and MET-minutes, and baseline value was added as fixed factor.

**Table 1:** Baseline characteristics overall and of participants in CLI and CLI+. Data collected in the Netherlands, 2010–2015.

	Overall (n = 411)	CLI (n = 164)	CLI+ (n = 247)
Age (in years)	55 ± 12	54 ± 12	56 ± 12
Sex (% Male)	35	36	35
BMI (in kg/m2)	34.5 ± 4.4	35.0 ± 4.5	34.2 ± 4.2
Waist circumference (cm)	113.1 ± 11.2	113.5 ± 11.3	112.8 ± 11.1
Diabetes (% Yes)	38	34	41
MVPA time (minutes/week)*	300 ± 395	249 ± 317	335 ± 438
Walking time (minutes/week)	217 ± 281	199 ± 238	229 ± 307
Total physical activity (METminutes/week)*	2342 ± 2675	1964 ± 2040	2591 ± 3000
Sitting time (minutes/day)	391 ± 191	385 ± 188	394 ± 194
Compliance with physical activity norm (%)	52	52	53
HbA1c (mmol/mol)			
Participants with diabetes	54.7 ± 14.7	54.5 ± 15.3	54.8 ± 14.4
Participants without diabetes	39.3 ± 6.2	40.2 ± 6.6	38.3 ± 5.6
SBP (mmHg)	137 ± 17	139 ± 18	136 ± 16
DBP (mmHg)	84 ± 10	85 ± 11	84 ± 10

Data are percentage or mean ± sd. BMI = Body Mass Index; MVPA = moderate to vigorous physical activity; SBP = systolic blood pressure; DBP = diastolic blood pressure.

*Significant difference between CLI and CLI+ (p < 0.05).

### 3.2 Drop-out and loss to follow-up

**Fig. 1:**
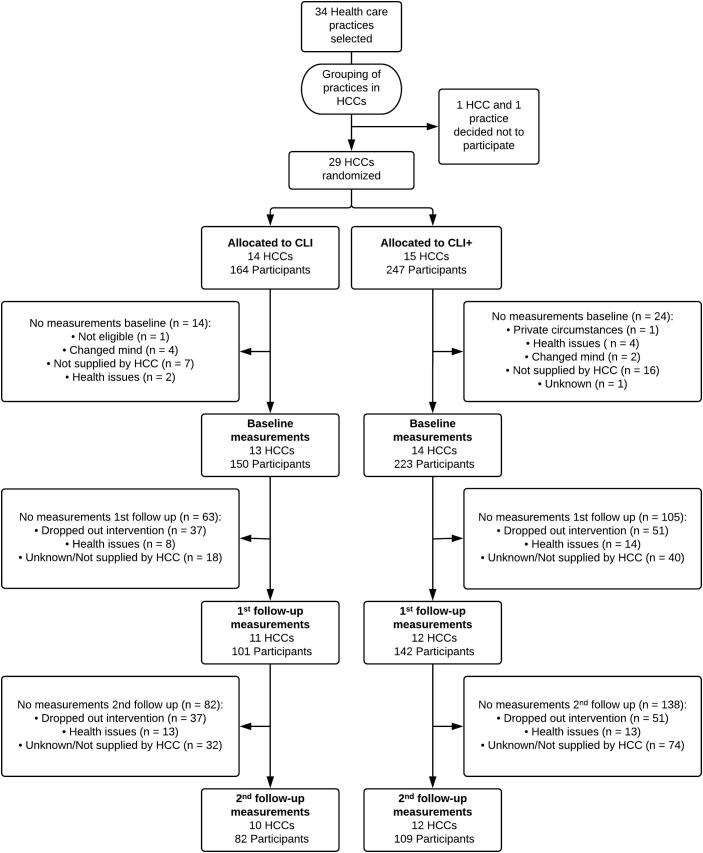
Flow diagram of recruitment of health care clusters (HCC) and participants, drop-out and annual measurements. Data collected in the Netherlands, 2010–2015.

Of 411 participants, a total of 89 participants (22%) did not complete the 12-month intervention period (Fig. 1). Chi-square tests showed that percentage of drop-outs did not differ between the two programmes (p = 0.643) or for other baseline characteristics. Sixteen participants (five in CLI and 11 in CLI+) dropped out immediately after recruitment, because the HCC was unable to start up the study (n = 3), recruitment mistakes (n = 2), health issues (n = 1) and unknown (n = 10). Within the 12 months of guidance, 73 participants dropped out, of which 33 in CLI and 40 in CLI+. Reasons for dropping out were health issues (i.e. (serious) adverse events, n = 27), unknown (n = 17), private circumstances (n = 9), lack of time, not motivated or wrong expectations (n = 13), moved (n = 4), financial issues (n = 2), and fear of PA (n = 1).

Within the 24 months of follow-up, 26 serious adverse events were registered (18 in CLI and 8 in CLI+; e.g. diagnosis of cancer or cardiovascular disease). Two participants were excluded from analyses due to pregnancy. The incidence of (serious) adverse events was not different between the programmes.

### 3.3 Physical activity

Mixed models showed no between-group differences between CLI and CLI+ in PA measures and sitting time (p = 0.221–0.869; Table 2), and neither was there interaction between the programmes and moment of measurement (p = 0.386–0.993). Within-group changes over time were significant: In both CLI and CLI+ walking time increased compared to baseline at 12, 18 and 24 months with respectively 88 ± 28, 106 ± 31 and 104 ± 29 min per week (p = 0.008; p = 0.002 and p = 0.001, respectively). After six months, daily sitting time had decreased with 43 ± 14 min (p = 0.008). No within-group changes over time were observed in weekly minutes of MVPA and total PA level (p = 0.165 and p = 0.102). Overall, at 12 and 24 months, 54.7% and 52.5% of participants complied with the Dutch PA norm (no significant change from baseline). At 12 and 24 months, 77.1% and 93.9% of participants reported to have adopted an independent physical exercise activity. The percentage of participants who complied with the Dutch PA norm and who adopted an independent activity at 12 and 24 months did not differ between programmes (p = 0.124 and p = 1.000).

**Table 2:** Differences in lifestyle over time and between CLI and CLI+. Data collected in the Netherlands, 2010–2015.

	N		6 months	12 months	18 months	24 months	p-value interaction	p-value CLI vs. CLI+	p-value time
MVPA time (minutes/week)	242	CLI	16 ± 52	101 ± 44	115 ± 47	160 ± 52	0.663	0.689	0.165
		CLI+	−10 ± 41	−10 ± 42	14 ± 47	−7 ± 41			
Walking time (minutes/week)	240	CLI	64 ± 31	112 ± 36*	109 ± 34*	101 ± 38*	0.937	0.835	0.000
		CLI+	51 ± 54	69 ± 32*	118 ± 40*	83 ± 35*			
Total physical activity (METminutes/week)	206	CLI	208 ± 340	829 ± 278	901 ± 329	911 ± 316	0.386	0.869	0.102
		CLI+	68 ± 298	284 ± 293	676 ± 305	311 ± 323			
Sitting time (minutes/day)	248	CLI	−62 ± 18*	−7 ± 24	−36 ± 22	−12 ± 23	0.993	0.221	0.021
		CLI+	−40 ± 18*	−16 ± 18	−35 ± 20	−11 ± 19			
Fruit consumption (pieces/week)	256	CLI	0.7 ± 0.7*	0.4 ± 0.7*	1.5 ± 0.7*	0.8 ± 0.7*	0.541	0.391	0.003
		CLI+	2.0 ± 0.4*	1.8 ± 0.5*	1.4 ± 0.5*	1.6 ± 0.6*			
Vegetable consumption (table spoons/week)	253	CLI	2.6 ± 1.2	1.1 ± 1.3*	3.6 ± 1.5*	1.0 ± 1.4	0.056	0.904	0.001
		CLI+	0.8 ± 1.1	4.2 ± 1.3*	3.0 ± 1.3*	0.8 ± 1.0			
Snack consumption (pieces/week)	255	CLI	−0.5 ± 0.2	−0.3 ± 0.2*	−0.6 ± 0.2*	−0.3 ± 0.2*	0.707	0.876	0.001
		CLI+	−0.2 ± 0.2	−0.5 ± 0.2*	−0.5 ± 0.2*	−0.6 ± 0.2*			
Candy consumption (pieces/week)	256	CLI	−1.3 ± 0.4*	−1.0 ± 0.5*	−0.7 ± 0.4*	−0.2 ± 0.5	0.511	0.134	0.000
		CLI+	−0.7 ± 0.3*	−1.1 ± 0.4*	−1.2 ± 0.4*	−0.7 ± 0.3			

Data are mean ± SE. MVPA = moderate to vigorous physical activity.

*Significantly different from baseline.

### 3.4 Diet

No between-group differences were detected in dietary behaviour (p = 0.134–0.904; and for interaction p = 0.056–0.707). Consumption of fruit was increased at all moments within-groups compared to baseline, ranging from an average increase of 1.1 ± 0.4 to 1.6 ± 0.4 pieces per week (p-values ranging from 0.001 to 0.036). Weekly vegetable consumption increased within-groups at 12 and 18 months compared to baseline with respectively 3.4 ± 0.9 and 3.0 ± 0.9 table spoons (p < 0.001 and p = 0.002). Intake of fatty snacks within-groups decreased compared to baseline at 12, 18 and 24 months, with 0.4 ± 0.1 to 0.5 ± 0.1 times per week (p-values ranging from 0.001 to 0.022). Weekly intake of candy decreased at six, 12 and 18 months with 1.0 ± 0.2 within-groups (all p < 0.001).

### 3.5 Cardiovascular risk factors

Waist circumferences showed significant between-group differences: The decrease in waist circumference at 12 months was 4.0 cm larger in CLI+, compared to CLI (p = 0.011), but this difference disappeared after 24 months (Table 3). Overall, waist circumference decreased with respectively 4.9 ± 0.7 cm and 4.2 ± 0.9 cm at 12 and 24 months compared to baseline (p < 0.001). Other health related outcomes did not differ between programmes (p = 0.067–0.828; and for interaction p = 0.106–0.602). Within-groups, BMI decreased with respectively 1.1 ± 0.2 kg/m^2^ at 12 months and 1.0 ± 0.2 kg/m^2^ at 24 months compared to baseline (p < 0.001); fat percentage decreased with respectively 1.8 ± 0.4 and 2.3 ± 0.5 compared to baseline (p < 0.001). Also, HbA1c values were temporarily decreased after one year with 1.6 ± 0.6 mmol/mol (p = 0.019) within-groups. Creatinine, cholesterol and HDL levels did not change over time. Diastolic blood pressure changed favourably over time (p = 0.047), but pairwise analysis between 0, 12 and 24 months were not significant. Systolic blood pressure was on average 4.3 ± 1.3 mmHg lower at 12 months compared to baseline (p = 0.001). However, after 12 months of follow-up this effect on systolic blood pressure had disappeared.

**Table 3:** Differences in health parameters over time and between CLI and CLI+. Data collected in the Netherlands, 2010–2015.

	N		12 months	24 months	p-value interaction	p-value CLI vs CLI+	p-value CLI vs CLI+
BMI (in kg/m^2^)	218	CLI	−0.8 ± 0.3*	−0.7 ± 0.3*	0.458	0.821	0.000
		CLI+	−1.2 ± 0.2*	−1.5 ± 0.3*			
Weight (kg)	229	CLI	−2.5 ± 0.7*	−1.2 ± 0.8*	0.379	0.531	0.000
		CLI+	−3.7 ± 0.7*	−3.8 ± 0.8*			
Waist circumference (cm)	192	CLI	−2.9 ± 1.0*	−3.1 ± 1.5*	0.011	0.347	0.000
		CLI+	−6.9 ± 0.9*	−5.3 ± 0.9*			
Fat percentage (%)	209	CLI	−1.3 ± 0.4*	−1.3 ± 0.8*	0.576	0.828	0.000
		CLI+	−1.9 ± 0.4*	−2.7 ± 0.7*			
HbA1c (mmol/mol)	142	CLI	−1.8 ± 0.8*	0.2 ± 1.0	0.277	0.514	0.022
		CLI+	−2.5 ± 1.1*	0.0 ± 1.3			
Total cholesterol (mmol/L)	169	CLI	−0.06 ± 0.11	−0.06 ± 0.12	0.441	0.318	0.154
		CLI+	−0.29 ± 0.10	−0.19 ± 0.15			
HDL (mmol/L)	162	CLI	−0.12 ± 0.04	−0.05 ± 0.04	0.602	0.145	0.348
		CLI+	−0.10 ± 0.10	−0.13 ± 0.18			
SBP (mmHg)	213	CLI	−6.2 ± 1.8*	−1.8 ± 1.7	0.106	0.067	0.001
		CLI+	−1.2 ± 1.4*	0.4 ± 1.8			
DBP (mmHg)	214	CLI	−2.9 ± 1.3	−2.5 ± 1.2	0.294	0.180	0.047
		CLI+	0.5 ± 1.0	−0.7 ± 1.1			

Data are mean ± SE. BMI = Body Mass Index; SBP = systolic blood pressure; DBP = diastolic blood pressure. *Significantly different from baseline.

### 3.6 Per protocol analyses

In the exploratory per protocol analyses (N varied between 130 and 255) there were no significant differences between the programmes. With regards to within-group changes, the difference in walking time between 24 months and baseline was not significant and sitting time was not significantly decreased at six months. Diastolic blood pressure decreased in both programmes significantly at 24 months, compared to baseline (p = 0.048). Other findings were similar to the intention to treat analyses.

## 4 Discussion

This study revealed that participants showed beneficial changes in lifestyle behaviours as well as in cardiovascular risk factors after participating in both a standard CLI and a high dose CLI+. The additional physical exercise group sessions supervised by a physiotherapist in CLI+ did not cause sustained enhanced benefits compared to the standard CLI. We conclude that the higher amount of PA guidance by the physiotherapist did not lead to additional effects compared to a standard CLI.

Evidence regarding the optimal amount and type of guidance in primary care is incomplete. The extra group sessions in CLI+ were hypothesised to lower barriers towards adopting independent exercise activities via feelings of competence and relatedness, two factors promoting intrinsic motivation as proposed by the Self-Determination Theory (Deci and Ryan, 2000). The earlier published process evaluation revealed that the number of attended group sessions was lower than planned, but that CLI+ was still substantially more elaborate than in CLI and the group sessions were especially valued by participants (Berendsen et al., 2015). However, the same evaluation revealed that the addition of group sessions might conflict with feasibility in real-life practice, as participants in CLI+ compensated the higher amount of guidance in groups by attending fewer individual meetings. As the individual meetings were deemed essential for setting individual and realistic goals via MI (Helmink et al., 2010, Lundahl et al., 2013), participants in CLI+ might have been less supported to set realistic and personal goals. A systematic review regarding lifestyle interventions in primary care revealed that interventions with more sessions resulted in greater weight loss (Leblanc et al., 2011), but did not take into account whether sessions consisted of evidence-based behavioural change techniques. Another systematic review suggested that a single, longer session of MI would be preferred for behaviour change instead of many sessions, as the total amount of time in MI was related to outcomes, but the total number of sessions was not (Lundahl et al., 2013). In response to controlled studies with high amount of guidance (Knowler et al., 2002, Goodpaster et al., 2010, Tuomilehto et al., 2001), earlier findings from the current study indicated that the combination of two or three group sessions per week with regular individual meetings was not realistic in daily primary care (Berendsen et al., 2015). Interestingly, the conclusion of the current study does not stand alone: Another primary care based study showed no evident dose–response relationship between the attendance of counselling sessions and clinical outcomes (Hardcastle et al., 2008). In short, lifestyle counselling in primary care can be effective and feasible, but adding multiple group sessions does not necessarily lead to additional benefits.

Overall, minutes of MVPA did not change over time after participation in CLI or CLI+; nevertheless, self-reported walking and sitting time, body composition and cardiovascular risk factors did improve, confirming the effectiveness of CLI in general. After the intervention period of one year and the follow-up at two years, weight had decreased with 3.4% and 2.7% respectively compared to baseline, revealing a sustained weight loss after termination of guidance by HCPs. The lack of a control group receiving no intervention does not allow causal conclusions about the effectiveness of CLI and CLI+, but results are in line with observational studies regarding effects on lifestyle and motivation of this specific CLI in primary care (Rutten et al., 2014, Helmink et al., 2011). Comparable lifestyle interventions have also shown positive results with regard to body composition, although the magnitude of changes differs between studies (Knowler et al., 2002, Look Ahead Research Group, 2010, Tuomilehto et al., 2001, Goodpaster et al., 2010, Hardcastle et al., 2008, Vermunt et al., 2012, Driehuis et al., 2012, van Rinsum et al., 2018). The decreases in BMI and waist circumference in the current study were markedly larger than found in three earlier studies (Hardcastle et al., 2008, Vermunt et al., 2012, Driehuis et al., 2012). These studies either consisted of fewer sessions than our CLI (Hardcastle et al., 2008) or the population had lower BMI at baseline (Vermunt et al., 2012, Driehuis et al., 2012), decreasing the potential for effects. In contrast, several other intervention studies reported larger effects on weight loss (Knowler et al., 2002, Look Ahead Research Group, 2010, Goodpaster et al., 2010, Tuomilehto et al., 2001). These studies comprised a population with higher BMI (Knowler et al., 2002, Goodpaster et al., 2010, Tuomilehto et al., 2001) or with diabetes (Look Ahead Research Group, 2010) and were executed in a controlled experimental setting (Knowler et al., 2002, Goodpaster et al., 2010, Tuomilehto et al., 2001), which might have increased the opportunity for effects compared to our interventions which were integrated in daily primary care. The Look AHEAD study showed that weight regain after the initial intervention period was diminished due to long term guidance (Look Ahead Research Group, 2010, Wadden et al., 2011), suggesting that sustained guidance might be necessary to sustain effects. The CLI in the current study involved guidance for one year only; the findings at two years indicate that participants were able to maintain several, but not all, beneficial outcomes without long term guidance. Therefore, it is encouraging that especially in this real-world setting, effects of the CLI can be sustained during follow-up.

The decrease in waist circumference, and the combination of increased self-reported walking time and decreased sitting time are of importance, given their beneficial effects on mortality and the development of diabetes mellitus (Bankoski et al., 2011, van der Ploeg et al., 2012, Duvivier et al., 2013). Several lines of evidence indicate that sedentary behaviour is an independent risk factor for cardiovascular disease and mortality (Koster et al., 2012, Ekelund et al., 2016). Previous studies observed that when a lifestyle intervention is aimed at increasing energy expenditure with more MVPA, total energy expenditure may not rise due to a compensatory decrease in light PA (Driehuis et al., 2012) and/or increase in sedentary time (Helmink et al., 2013). This compensatory mechanism can be counteracted by including strategies that aim to increase non-exercise PA and reduce sedentary time. Paying more attention to such strategies in future CLI might improve the long-term health benefits in addition to the benefits of increased PA.

The prospective design of the current study is one of its strengths; also, the inclusion of objectively measured health parameters, such as BMI, fat percentage and other cardiovascular risk factors strengthen our conclusions. The study was performed in primary care practices with local HCPs; the pragmatic design and setting maximises generalisability of our findings to daily practice. This probably also led to a more flexible execution of the intervention which can contribute to a type III error, with a higher loss to follow-up compared to trials with a controlled experimental setting (Knowler et al., 2002, Tuomilehto et al., 2001). Specifically, the study showed a large loss to follow up of accelerometer measurements, due to a.o. questionable user friendliness of the used device (Berendsen et al., 2015), and the IPAQ was used to assess PA instead. Short food frequency questionnaires are feasible in practice and quality of certain aspects were previously reported as acceptable, but measuring multiple aspects of diet with short questionnaires cannot bring definite conclusions. We provided and collected questionnaires via mail to minimise social desirability. Nevertheless, the recruitment did not meet the calculated sample size, so there might be a lack of power. Post-hoc calculations revealed small effect sizes, indicating that differences in MVPA between the programmes were trivial, supporting the conclusions based on the p-values of the multilevel analyses.

## 5 Conclusion

Excess body weight is an important cause of increased risk for non-communicable diseases and high health care costs. The current study adds to existing literature, indicating that a combined lifestyle intervention is effective in decreasing cardiovascular risk factors, via MI and goal setting with a team of HCPs in primary care (LSC, physiotherapist and dietician). Participants in both programmes showed sustained beneficial changes in PA behaviour and diet compared to baseline, accompanied with sustained decreased BMI, waist circumference and HbA1c. No differences were found between CLI+ and the standard CLI, revealing that adding group sessions aimed at experiencing PA and initiating group cohesion does not seem to lead to sustained additional health benefits. Thus, a standard CLI, consisting of six individual meetings with the LSC, ten meetings with dietician, and six individual meetings with physiotherapist would be sufficient to facilitate a healthy lifestyle and improve health in a population with high weight related health risk.

## CRediT authorship contribution statement

**Brenda A.J. Berendsen:** Investigation, Formal analysis, Writing - original draft, Visualization. **Marike R.C. Hendriks:** Investigation. **Geert M. Rutten:** Investigation. **Stef P.J. Kremers:** Conceptualization, Methodology, Funding acquisition. **Hans H.C.M. Savelberg:** Conceptualization, Methodology, Funding acquisition, Supervision. **Nicolaas C. Schaper:** Conceptualization, Methodology, Funding acquisition, Supervision.

## Declaration of Competing Interest

The authors declare that they have no known competing financial interests or personal relationships that could have appeared to influence the work reported in this paper.
